# Application of Tissue Engineered Nanomaterials in Meniscus Sports Injury Repair

**DOI:** 10.3389/fbioe.2022.905869

**Published:** 2022-06-14

**Authors:** Yan Han

**Affiliations:** College of Physical Education, Xuchang University, Xuchang, China

**Keywords:** tissue engineering nanomaterials, meniscus repair, Kalman filter theory, KOA exercise therapy, sports injury

## Abstract

In daily life and sports activities, the knee joint is the dominant joint. Movements such as walking upstairs, running, and walking require the knee joint to function. The principle of tissue engineering and the technical methods of molecular biology to construct functional meniscus replacement products *in vitro* have become an ideal method to fundamentally solve the meniscus injury. This paper aims to study the application of tissue engineered nanomaterials in meniscal sports injury repair. In this paper, KOA exercise therapy based on Kalman filter theory is proposed, which has a great effect on the rehabilitation of bone tissue injuries. The experimental results of this paper show that in the number of people with meniscus injuries in 2013, the percentage of people younger than 25 years old was 13%, and the percentage of people younger than 25 years old in 2020 was 12%, which did not change much. However, the percentage of people over 40 years old was 57% in 2013, and by 2020, the percentage is 66%. Although the increase is not large, the percentage of people over the age of 40 is the highest every year, which means that older people have a higher proportion of meniscal injuries.

## Introduction

The knee meniscus is an important part of the structure and function of the knee joint. It not only has the function of supporting and distributing the load, but also maintaining the stability of the joint. Meniscus damage is related to joint instability and load transmission barriers, and even the occurrence of joint deformity arthrosis.

With the advancement of science and technology and the continuous exploration of complications in the repair of meniscus damage, the current method of repairing damaged meniscus is mainly transplantation or repair to maintain the function of the meniscus, but the effect is not very good. With the development of tissue engineering technology and the emergence of tissue scaffolds, a new treatment method has been provided for repairing meniscal tissue damage. The development of ideal meniscal scaffold materials has become a hot research topic for scholars at this stage.

The innovation of this paper is that: 1) Introduce the theoretical knowledge of tissue engineering nanomaterials and meniscus sports injury repair. And the KOA exercise therapy based on Kalman filter theory was used to analyze the importance of tissue engineered nanomaterials in the repair of meniscus sports injuries. 2) The theory of Kalman filter and the method of tissue engineering nanomaterials are expounded. Through experimental research, it was found that tissue engineering nanomaterials can promote the speed of meniscus sports injury repair.

## Related Work

With the development of sports in recent years, people pay more and more attention to sports. Waldrop N E found that the number of toe injuries has been increasing in recent years. Plantar restraint injuries to the toe joints can cause significant disability in athletes, affecting their ability to perform on the sports field. Most toe injuries can be managed conservatively with rest, ice, compression, immobilization if necessary, and a dedicated rehabilitation program. However, in some injuries, the plantar joint is torn and the joint becomes unstable. If necessary, the damage can be repaired with surgery. However, the scholar has no specific experimental subjects to prove whether the injury can be treated conservatively (Waldrop, 2021). Song H found that in recent years, with the advancement of computer technology, many information and image evidences in the medical field are being developed. At present, sports medical data is indispensable to the medical sector, which utilizes massive medical data and events for reliable interpretation. And it has become an important research path for medical data collection and analysis. Song H discussed the extraction, research, and lack of training and accuracy of complex algorithms for critical sports medicine data. However, the scholar did not draw specific conclusions (Song et al., 2021). In Mmfa B surveyed in patients and athletes of all ages, physical activity is considered an active lifestyle with positive effects on healthy aging, which unfortunately leads to higher rates of sports injuries. In sports, where early and accurate injury diagnosis is critical for early treatment to achieve full recovery, imaging technologies are increasingly important for the successful diagnosis and management of patients. Why imaging technology is becoming more and more important, the scholar did not describe the advantages of imaging technology (Font, 2020). Robotti G found that in recent years, women’s sports participation has increased and women’s sports have become more and more challenging. Although more women are participating in sports, there is still a lack of information on sex-specific lesions. Robotti G therefore assessed gender differences in sports injuries, and understanding gender-specific injuries and injury mechanisms is important to assess the correct diagnosis. But the scholar did not explain how he assessed gender differences in sports injuries (Robotti et al., 2020). Trentacosta N found that with increasing participation of children in organized sports and children’s early specialization in individual sports, the number of sports injuries occurring in the pediatric and youth sports populations is also increasing. Children experience acute traumatic injuries during practice and competition. The uniqueness of pediatric patients often requires a different diagnosis, prognosis, and treatment approach for sports injuries compared to adult patients. However, the scholar did not clearly describe what the different diagnosis, prognosis and treatment methods are (Trentacosta, 2020). Bulat M found that musculoskeletal simulation and dynamic modeling procedures have been used to gain insight into lower extremity musculoskeletal injury mechanisms. In addition to spatiotemporal, kinematic, and kinetic data obtained from motion analysis systems, musculoskeletal simulation programs can provide information on joint contact and muscle forces, as well as levels of muscle activation. Musculoskeletal simulation platform may help assess risk factors for sports-related injuries. Injury prevention planning using musculoskeletal simulation may help reduce the incidence of sports injuries and allow for rapid recovery from injuries. However, the scholar did not have specific experimental objects and experimental data (Bulat et al., 2019). Joao B proposed that advanced strategies for bioengineering fibrocartilage tissue to restore meniscal function are necessary. Currently, 3D bioprinting technology has been used to fabricate clinically relevant patient-specific complex structures to meet unmet clinical needs. He characterized each bioink formulation by measuring rheological properties, swelling ratio, and compression mechanical behavior. The alignment of collagen fibers was achieved in a bioprinted hybrid structure. The results demonstrate that this bioprinted mechanically enhanced hybrid structure provides a versatile and promising alternative for the production of advanced fibrocartilage tissue (Costa et al., 2020).

## Koa Exercise Therapy Based on Kalman Filter Theory

**FIGURE 1:**
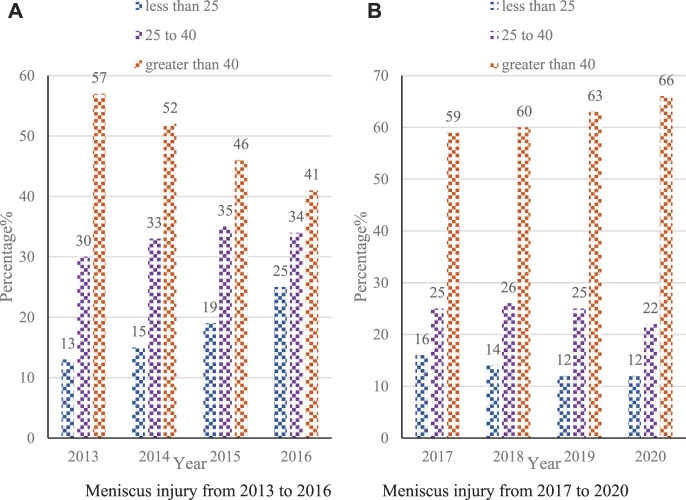
Age ratio of meniscal injuries from 2013 to 2020. **(A)** Meniscus injury from 2013 to 2016 **(B)** Meniscus injury from 2017 to 2020.

In cartilage tissue engineering, three-dimensional scaffolds serve as temporary extracellular matrix, providing a place for chondrocytes to attach, proliferate, differentiate and metabolize. An ideal cytoscaffold should have the following properties: three-dimensional structure and high porosity (interconnected pore network) to provide cell growth and transport of nutrients and excretion of metabolites. Controlled degradation rate and absorption rate. Suitable surface chemistry for cell adhesion, proliferation and differentiation. Match the mechanical properties of the corresponding tissue implant site, and nanomaterials have this property. Human meniscus injury is a very common disease, mostly caused by sports injuries (Boric et al., 2019). The meniscus is located in the middle part of the knee joint, which is very complex in structure, and bears various loads in daily life and sports activities, such as extrusion, rotation, stretching, shearing and so on. When the meniscus is contused, the normal function of the knee joint will be greatly weakened, and the knee joint will be seriously burdened, especially in strenuous physical activities (Borgstrom et al., 2019). Meniscal tissue vulnerability is more common in people under the age of 40 and people over the age of 60. The former is mainly caused by the coercive destruction brought about by exercise, and the latter is mainly caused by the degeneration of the organization function. According to incomplete statistics, about 60% of people over the age of 40 have the incidence rate. And this group is often accompanied by meniscus tearing and other phenomena as they grow older (Ding et al., 2022). The age ratio of meniscus injuries from 2013 to 2020 is shown in Figure 1:

Since the regenerative ability of the removed meniscus is extremely weak or even has no regenerative function. The lack of meniscus can cause very serious femoral condyle deformation, joint space tightening, osteophytes and other symptoms, and even lead to osteoarthritis. So people pay more and more attention to the meniscus injury (Hashimoto et al., 2018).

### Model Parameter Identification Based on EKF

Because of its simple and efficient algorithm, the Kalman filter algorithm is a linear unbiased optimal estimation algorithm based on the minimum mean square error when the system satisfies Gaussian white noise and linearity, so it is widely used in many fields (Falagan-Lotsch et al., 2017).

Consider a state-space model of the form; the state equation is as in Eq. (1):

$$A_{k-1}=\Phi_{k-1}+W_{k-1}$$ (1)

The observation equation is as Eq. (2):

$$Z_{k}=H_{k}A_{k}+V_{k}$$ (2)

$Z_{k}$ and $A_{k}$ are the system process noise sequence and the observation noise sequence, respectively. And it satisfies the independent Gaussian white noise with zero mean and variance $V_{k}$ , and the initial state is also independent of each other. It is assumed that the statistical characteristics of the system process noise and observation noise are as in Eq. (3):

$$E\left[w_{k},w_{j}^{T}\right]=Q_{k}\delta_{kj}$$ (3)

In the equation: $Q_{k}$ is the non-negative definite variance matrix of $w_{k}$ ; $\delta_{kj}$ is the symmetric positive definite variance matrix. The implementation steps of the Kalman filter algorithm are as shown in Eq. (4):

$$A_{0,0}=E\left[A_{0}\right]$$ (4)

Observation update (correction): Given a new observation value of $K_{k}$ , calculate the filtered mean and the estimated error variance matrix as in Eq. (5):

$$K_{k}=P_{k,k-1}H_{k}^{t}{\left[H_{k}P_{k,k-1}H_{k}^{t}+R_{k}\right]}^{-1}$$ (5)

**FIGURE 2:**
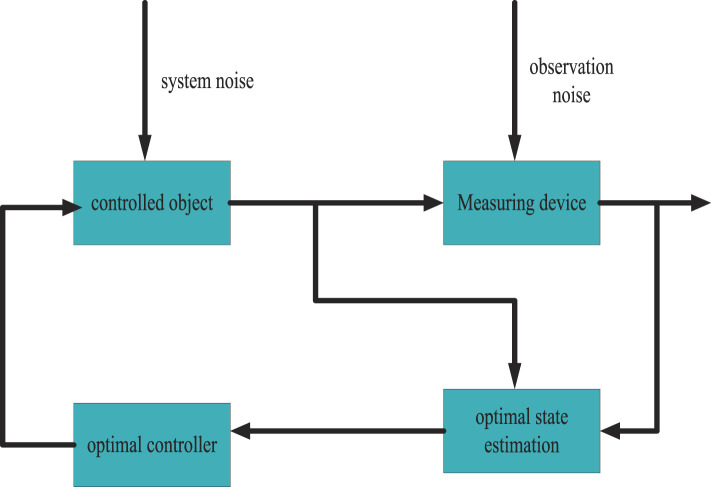
Kalman filter structure diagram.

Kalman filter is a linear and unbiased optimal state estimation algorithm based on the minimum mean square error criterion in linear systems, so it has been widely used in many fields (Mirkin et al., 2017). The structure diagram of Kalman filter is shown in Figure 2:

As shown in Figure 2: For general nonlinear systems, it is difficult to find a strict recursive filtering equation, and the approximate method is usually used to study it. So when the system is nonlinear, people can linearize the system at each time step. Thus transformed into a nonlinear system that approximates a linear time-varying system, then the Kalman filter is applied to the linear time-varying system. This is the idea of the extended Kalman filter for dealing with nonlinear systems (Deng et al., 2017; Li et al., 2022).

The derivation process of the extended Kalman filter algorithm is as follows. Consider the following nonlinear system state space model such as Eq. (6):

$$A_{k}=f\left(A_{k-1},k-1\right)+W_{k-1}$$ (6)

In the equation, $A_{k-1}$ and $W_{k-1}$ are the system process noise sequence and the observation noise sequence, respectively.

Expand the nonlinear function of the observation equation into a Taylor series around the filter value, retaining the first-order term and ignoring the second-order, as shown in Eq. (7):

$$Z_{k}=\left(A_{k,k-1},k\right)+\frac{\partial h}{\partial A_{k}}$$ (7)

Then the observation equation is Eq. (8):

$$Z_{k}=H_{k}A_{k}+b_{k}+v_{k}$$ (8)

Then combined with the basic equations of standard Kalman filtering, the implementation steps of the extended Kalman filtering algorithm can be obtained as Eq. (9):

$$P_{k,k}=\left[I-K_{k}H_{k}\right]P_{k,k-1}$$ (9)

In its recently updated rules, the EKF filtering algorithm uses only the first two order information mean and variance of the system equation. Although the extended Kalman filter algorithm has many advantages and is widely used in practical problems, the EKF is not perfect (Lee et al., 2017). It still has limitations. Although many scholars have proposed many improved algorithms based on EKF, these shortcomings are still not solved. Therefore, it is necessary to find a filtering algorithm that is more suitable for applying nonlinear systems (Duhan et al., 2017).

According to the implementation steps of the extended Kalman filter algorithm introduced, the state variables of the model are identified by using the EKF algorithm and the acquired acceleration time series (Mihail et al., 2018). The state equation and observation equation in this paper are Equation (10) and (11), respectively:

$$A_{k}=F_{k,k-1}A_{k-1}+W_{k-1}$$ (10)

$$Z_{k}=h\left(A_{k},k\right)+v_{k}$$ (11)

**FIGURE 3:**
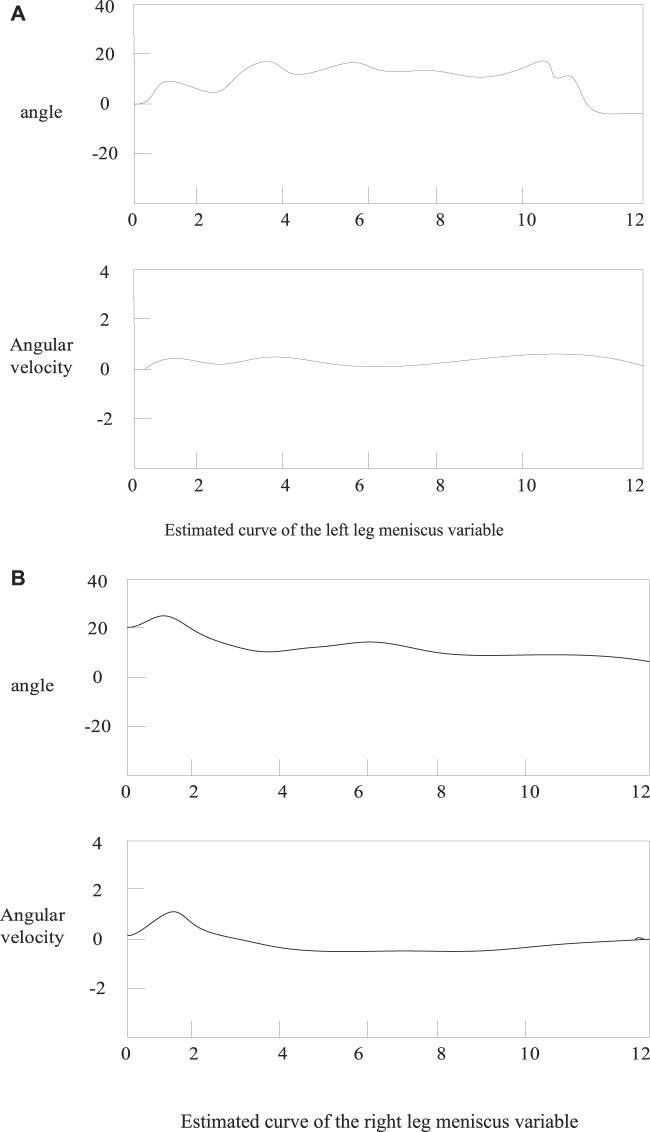
Meniscus variable estimation curve. **(A)** Estimated curve of the left leg meniscus variable **(B)** Estimated curve of the right leg meniscus variable.

The final state estimation error variance of the EKF algorithm is shown in Figure 3:

As shown in Figure 3: The static left thigh angle value converges to a certain value and the corresponding angular velocity value and angular acceleration value are almost zero. The estimation results of this model are consistent with the motion law of straight leg raising for knee joint motion (Silva et al., 2018; Stocco et al., 2022). At the same time, the variance of the state estimation error shown in the figure is very small, which means that the EKF can better estimate the state of the model from the observable short time series. These estimated motion state variables are then used as the basis for subsequent normative judgments (Valenzuela et al., 2019).

### Unscented KaIman Filter Calculation Process

The unscented Kalman filter is Equation (12) and (13):

$$A_{k+1}=f\left(A_{k},u_{k},W_{k}\right)$$ (12)

$$Z_{k}=h\left(A_{k},V_{k}\right)$$ (13)

In the equation, $A_{k+1}$ and c $Z_{k}$ are the process noise and observation noise of the system, respectively. The specific implementation steps of the unscented Kalman filtering method are as follows:

The parameters are calculated as Eq. (14):

$$\lambda =\alpha^{2}\left(n+k\right)-n$$ (14)

In Eq. (14): coefficient α is the main scale factor that determines the distribution range of Sigma points, and λ is a composite scale parameter.

Calculate the Sigma point, and bring the result obtained by the change of U into the Kalman basic equation, that is, the unscented Kalman filter equation is obtained. The steps are as in Eq. (15):

$$\xi_{k-1}^{\left(i\right)}=A_{k-1}^{a}$$ (15)

The time update equation is calculated, and through the propagation of the state equation, the Sigma point is calculated as in Eq. (16):

$$\xi_{k}^{\left(i\right)}=f\left(\xi_{k-1}^{\left(i\right)}\right)$$ (16)

The UKF algorithm and the acquired acceleration time series are also used to identify the state variables of the thigh system and calf system models. The UKF algorithm is used to identify the model state (Silva et al., 2018).

### Denoising Results

**FIGURE 4:**
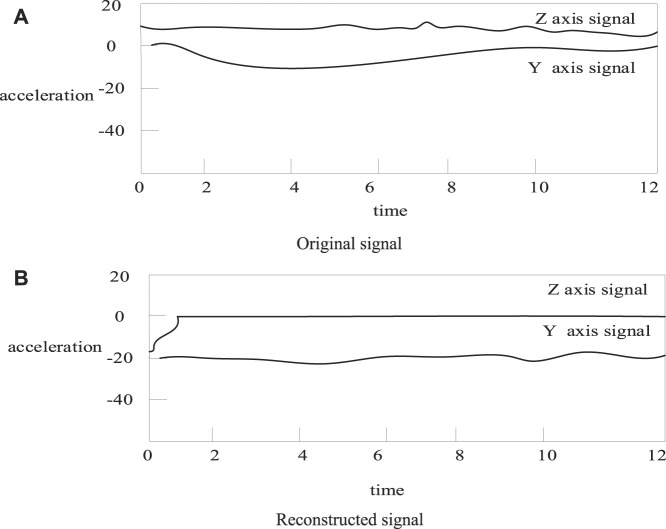
Signal comparison before and after extended Kalman filter. **(A)** Original signal **(B)** Reconstructed signal.

**FIGURE 5:**
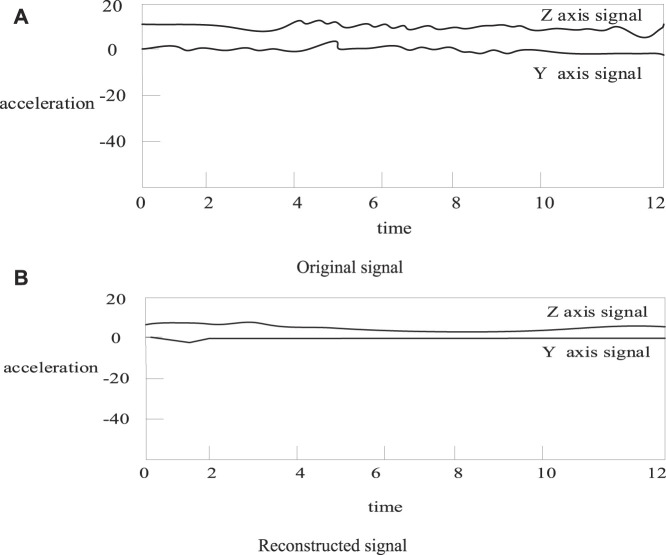
Signal comparison before and after unscented Kalman filtering. **(A)** Original signal **(B)** Reconstructed signal.

In this paper, the extended Kalman algorithm and the unscented Kalman algorithm not only estimate the state variables in the model, but also filter the acceleration signal (Stocco et al., 2022). In order to verify the denoising performance of the EKF and UKF algorithms for acceleration signals. The comparison chart is about calculating the signal before and after filtering by these two methods. As shown in Figures 4, 5:

As shown in Figures 4, 5: it can be seen that the filtered acceleration signal retains most of the energy of the original signal, and also effectively removes interference, and the filtering effect is good (Valenzuela et al., 2019). However, the filtering effect cannot be quantitatively evaluated from the figure. In order to quantify the filtering effect, the following equation is used to evaluate the filtering effect of EKF and UKF (Cnf et al., 2021). The smoothness SM is as Eq. (17):

$$SM=\frac{\sum_{K=2}^{N}{\left(A\left(k\right)-A\left(k-1\right)\right)}^{2}}{N}$$ (17)

In the equation: A(k) is the filter output value, SM is the smoothness index, which is used to calculate the smoothness of the filter output. The larger the smoothness index, the better the smoothness of the filtering output and the better the filtering effect. Deviation DV is as Eq. (18):

$$DV=\frac{\sum_{K=1}^{N}{\left(A\left(k\right)-B\left(k\right)\right)}^{2}}{N}$$ (18)

In the equation: A(k) is the filtered signal, B(k) is the original signal, and N is the deviation index, which is used to calculate the deviation between the filtered output value and the observed value. Under the condition of equal smoothness, the smaller the index value, the smaller the error after filtering. The signal-to-noise ratio SNR is as Eq. (19):

$$SNR=10*\log\frac{\sum_{K=0}^{N-1}{\left|B\left(k\right)\right|}^{2}}{\sum_{K=0}^{N-1}{\left|A\left(k\right)-B\left(k\right)\right|}^{2}}$$ (19)

In the equation: A(k) is the filtered signal, B(k) is the original signal, SNR is the signal-to-noise ratio index. The larger the index value, the higher the signal-to-noise ratio, which means the better the denoising effect. The filtering effects of the EKF and UKF methods are compared, and the results are shown in Table 1:

**TABLE 1:** Comparison of filtering effects of EKF and UKF methods.

Algorithm	EKF	UKF
SM for B	0.0081	0.0063
DV for B	0.2786	0.0042
SNR for B	0.009	0.0456
SM for Z	0.4053	0.0086
DV for Z	0.8957	0.6881
SNR for Z	4.4200	5.4065

It can be seen from Table 1 that when the smoothness of UKF is equivalent, the deviation index is small, the error after filtering is also small, and the signal-to-noise ratio of the UKF algorithm is high, and it has a good denoising effect. Therefore, the results show that the filtering effect of UKF is better than that of EKF.

This chapter mainly describes the processing of acceleration signals. First, the collected signals are preprocessed, and then the EKF and UKF are used to identify the model parameters of the processed human straight-footed motion data. Then the filtering effects of these two algorithms are analyzed.

## Properties of Tissue Engineered Nanomaterials

### Introduction and Development of Tissue Engineering Nanomaterials

**FIGURE 6:**
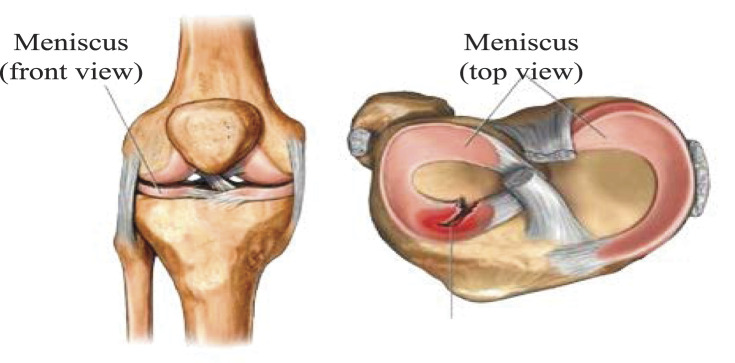
Meniscal fibrochondrocytes.

**FIGURE 7:**
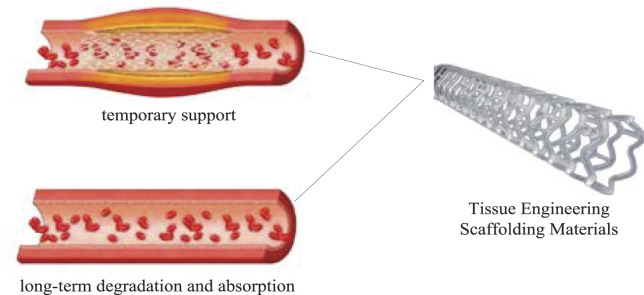
Tissue engineered nanomaterials.

Mitochondria, the main site of aerobic respiration to produce energy. The energy converters of plant cells are chloroplasts and mitochondria, and the energy converters of animal cells are mitochondria. Mitochondria can oxidize and decompose sugars in cells into carbon dioxide and water, and at the same time release chemical energy in organic matter for cells to use. In addition to providing energy for cells, mitochondria are also involved in processes such as cell differentiation, cell information transmission, and apoptosis, and have the ability to regulate cell growth and cell cycle. Tissue engineering nanomaterials are the key to bone tissue engineering. Tissue engineering nanomaterials, as important inorganic medical materials, have good osseointegration, biocompatibility and osteoconductivity, and become a research hotspot of bone repair materials. Carbon nanofibers (CNFs) have excellent mechanical properties and good biocompatibility, and are often used as reinforcing materials in bone tissue repair. Meniscal fibrochondrocytes are shown in Figure 6:

As shown in Figure 6: In recent years, with the continuous development of tissue engineering technology, it has brought new hope for the treatment of meniscus injury. Ideal seed cells for meniscal tissue engineering must possess the following properties: It has little damage to the body, can adapt to the pathological, physiological and biomechanical environment of the knee joint, and play the physiological function of the normal meniscus. The meniscal fibrocartilage cells can be directly obtained from the meniscus tissue of the joint. The tissue engineered nanomaterials are shown in Figure 7:

As shown in Figure 7: Nanomaterials developed in the early 1980s enjoy the title of “new materials beyond the century” with their unique physical and chemical properties and broad application prospects. Due to the unique structure of nanomaterials, it has four major effects: small size effect, surface effect, macroscopic quantum tunneling effect and quantum size effect. Nanoparticles have unique physical and chemical properties and can be used in optical, electrical, biotechnology, drug delivery, catalysis, magnetic materials and other fields.

The basic principle is that the osteoblasts cultured *in vitro* are seeded on bone tissue engineering materials, and then the composite of cells and transplant materials is implanted into the matrix bone defect to achieve bone tissue repair and regeneration. Among them, bone tissue engineering materials can promote cell proliferation, differentiation, secretion of ECM, and support the growth of new bone tissue, and finally can be degraded or absorbed by the body. The proposal and development of tissue engineering technology has brought a new dawn to the research of bone regenerative medicine. This avoids the drawbacks of traditional treatments and has important implications for clinical applications.

### Types of Tissue Engineering Scaffolds

(1) Metal material

In the 1920s, metal materials were quickly used in surgical operations due to their easy processing and low cost, mainly for the repair and replacement of hard tissues. The most widely used ones are stainless steel 316L, cobalt alloy and titanium alloy Ti-6A1-4V. The high mechanical strength and good corrosion resistance of these materials enable them to meet the mechanical stability requirements of bone graft materials. However, the limitation of metal materials is that the surface lacks biorecognition and is difficult to degrade, and also releases toxic metal ions due to wear, which leads to inflammation at the tissue site.

(2) Bioceramic materials

Bioceramic materials have a wide range of applications in surgical plastic surgery, bone tissue and tooth restoration. Because of its high strength and good chemical stability, it has been widely used in artificial joint replacement and oral ceramic restoration. However, it still has the disadvantage that the Young’s modulus is too high and causes local stress concentration in the bone tissue. Therefore, researchers compound bioceramic materials with other materials to improve the overall performance.

(3) Natural materials and polymer materials

Biomaterials from natural resources such as cellulose and chitosan have excellent biocompatibility, but they also have the disadvantages of insufficient mechanical properties and immune rejection. It is often combined with synthetic polymers or inorganic materials to improve its mechanical properties. Compared with natural materials, polymer materials have good mechanical properties and can design the morphology and size.

### Carbon Nanofibers

Since the use of carbon materials in 1960, there has been a great interest in carbon materials for biomedical applications such as biosensors, spinal cages, and human prostheses. Carbon nanofibers (CNFs) with diameters in the submicron and nanometer range exhibit excellent mechanical properties, high specific surface area, excellent electrochemical performance, and good biocompatibility. Among them, the elastic modulus of carbon nanofibers is very close to that of bone, and it is often used as a reinforcing material in bone tissue engineering.

**FIGURE 8:**
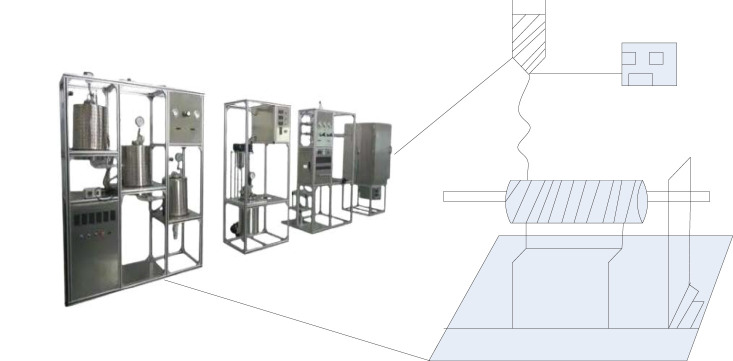
Equipment diagram of electrospinning device.

Among the preparation methods of CNFs, electrospinning is more simple and feasible than other traditional methods such as spraying method, chemical vapor deposition method, etc. It is very suitable for preparing carbon nanofiber membranes with high specific surface area, aspect ratio, certain porosity and excellent mechanical properties. The equipment diagram of the electrospinning device is shown in Figure 8:

As shown in Figure 8: Electrospinning is a facile and continuous technique for the preparation of nanofibers. Its main principle is to rely on the electrostatic repulsion between the surface charges of the polymer viscous fluid. The diameter of nanofibers prepared by electrospinning can reach tens of nanometers, which can be used to prepare polymer materials, ceramics, composite materials, etc. There are many factors affecting the preparation of composite nanomaterials by the combination of electrospinning technology and sol-gel method.

### Polymers for Preparing Carbon Nanofibers

**FIGURE 9:**
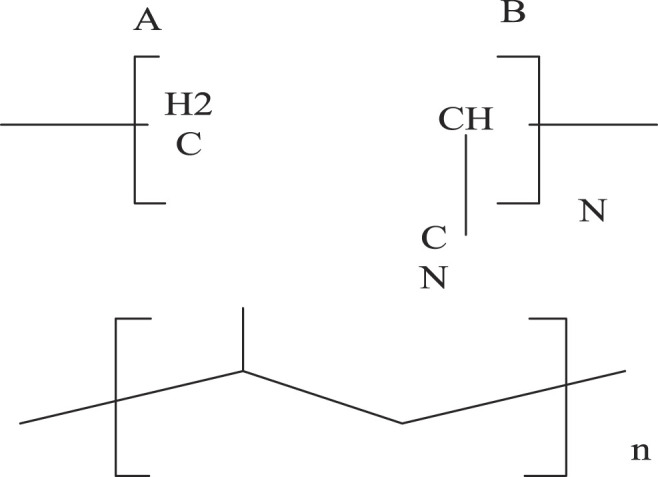
Chemical structure of PAN.

Polyacrylonitrile (PAN) and polyvinylpyrrolidone (PVP) are common polymers for the preparation of CNFs. PAN has a high melting point. Due to the strong intermolecular force of the PAN structure, the CNFs precursor has good thermal stability and is not easy to melt during the heat treatment process. Therefore, the obtained CNFs have relatively compact structure and high carbon yield. The chemical structure of PAN is shown in Figure 9:

As shown in Figure 9: PAN is a water-soluble linear polymer, easily soluble in most organic solvents, and has good biocompatibility. When preparing BG/polymer composites, the polymer needs to have hydrogen bond acceptors. The strongly polar amide group in PAN and the carbonyl group in PVP can act as hydrogen bond acceptors. It can form hydrogen bonds with SiO2 surface residues, so as to obtain a homogeneous mixed system of the two.

### Application of Carbon Nanofiber Composites in Bone Tissue Engineering

CNFs are selective for cell adhesion, smaller in diameter. CNFs can promote the adhesion of osteoblasts, but have no effect on the adhesion of fibroblasts, chondrocytes and smooth muscle cells. The precursor spinning solution was prepared by blending PAN-PMMA, and PMMA was decomposed during the heat treatment to form a slender porous structure. This morphological structure improves the specific surface area, roughness and porosity of the composites, thereby accelerating the dissolution and release rate of BG nanoparticles and promoting the proliferation and mineralization process of osteoblasts.

In the preparation process of the meniscus stent, in addition to the selection of materials, the molding process of the stent material also plays a crucial role in the performance of the stent. Whether it is the overall shape or the formation of the microporous structure or the degradation rate of the scaffold, in addition to the selection, testing, and analysis of scaffold materials, it is also necessary to have an in-depth understanding of their fabrication processes. Commonly used process methods are:

(1) Solution casting/particle leaching method

This method is relatively common in the preparation technology of tissue engineering scaffolds. The principle is to melt or dissolve the polymer scaffold material to be prepared into a liquid state, and add a pore-forming agent with the same size as the desired pore size. Under certain conditions, keeping the state of the polymer material unchanged, the pore-forming particles are dissolved and leached out, thereby forming a porous scaffold material. This method has certain limitations, such as limited stent thickness and poor pore connectivity.

(2) Thermally induced phase separation/freeze drying method

These two methods are generally used in combination. The principle is that the two-phase solution separates as the temperature drops, and the polymer-rich phase is frozen to remove the solvent to obtain a microporous membrane or scaffold. This method has certain limitations, its pore size is not easy to control, and the hyaluronic acid scaffold has good pore connectivity and is relatively uniform.

(3) Gas foaming method

The gas foaming method can effectively avoid the residue of organic solvents or particles caused by the introduction of toxic solvents. Using gas as a porogen, a scaffold material with a porosity of 80% and a pore size ranging from 30 to 100 μm can be obtained. However, the scaffold prepared by this method has a closed-cell structure. It has been analyzed that ultrasound can unclog the occluded pores of the stent, and good results have been achieved.

(4) 3D printing technology

Since the development of three-dimensional printing technology (3D printing), it has been widely used in the preparation of tissue engineering scaffolds. By scanning the anatomical shape of the damaged part of the tissue and editing it into computer language, a scaffold material with a specific shape is printed. Some scientists used the cell mixture as a bioink, supplemented by ultraviolet light irradiation, and 3D printed the cartilage defect site, and successfully printed the same defect cartilage model as *in situ*. 3D printing is currently an ideal technology for preparing tissue engineering scaffolds, but the cost is high.

## Experiments and Analysis of Meniscus Injury Repair

### Grouping and Experimental Methods of Meniscus Injury

Exosomes are tiny nanocapsules secreted by most cell types. The idea behind the coating is two-fold: First, because exosomes are composed of materials not too different from cell membranes, they “camouflage” the scaffold to trick smooth muscle cells and the body’s immune system. Preparation of Fibrochondrocyte-Fibrin Gel Complex Using fibrin gel as a scaffold material for tissue engineering meniscus, and using rabbit meniscus fibrochondrocytes as seed cells to prepare fibrochondrocyte-fibrin gel complexes.

Metabolic rate refers to the energy consumed by the human body per unit time. According to the type of energy metabolism, it can be divided into three types: basal metabolic rate, resting metabolic rate and active metabolic rate. In the article, the metabolic rate usually refers to the basal metabolic rate.

Eight white rabbits were numbered and randomly divided into two groups, A and B. Four in group A, four in group B. The knee meniscus in group A only caused full-thickness meniscus injury without any implantation, while in group B, fibrin gel was simply implanted in the knee meniscus defect.

**FIGURE 10:**
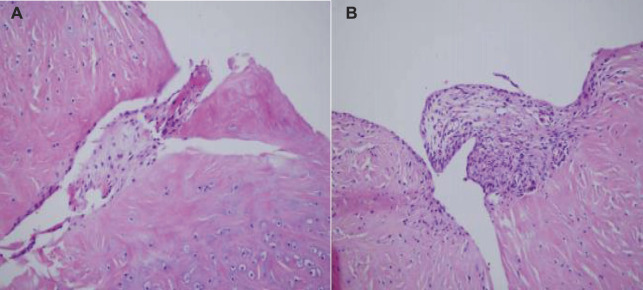
Histological observation 4 weeks after surgery (The picture is from my experiment).

By repairing the meniscus damage, the meniscus becomes a stable tissue that not only relieves clinical symptoms, but also protects articular cartilage and prevents the occurrence of traumatic arthritis. From the test results, fibrochondrocytes and fibrin gel complexes have a good repair effect on meniscus damage. The histological observation 4 weeks after surgery is shown in Figure 10:

As shown in Figure 10, the experiment was divided into group A and group B, group A represents four rabbits without any implants 4 weeks after the observation, and group B represents four rabbits with knee meniscus implanted. These 4-week specimens showed that the meniscus rupture in group A did not heal, the chondrocytes at the edge of the crack were inactive, and the metabolism was very low. In group B, however, the defective area was filled with fibrous tissue, and the cells were fibroblasts and numerous

**FIGURE 11:**
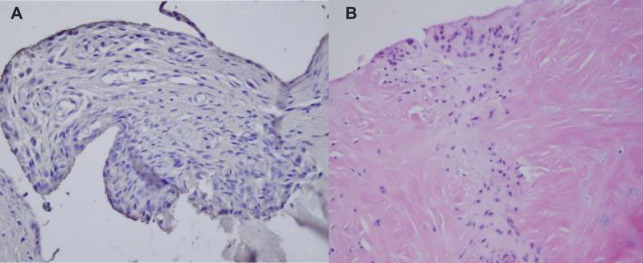
Histological observation 6 weeks after surgery (The picture is from my experiment).

As shown in Figure 11: The 6-week samples showed that the meniscal fissure in group A was incurable, and the chondrocytes at the edge of the fissure were in a state of inactivity and hypometabolism. In group B, the meniscus was more organized, and the collagen fibers were irregularly arranged, so that multiple chondrocytes were found to form cartilage capsules. The surrounding normal meniscal tissue is similar and has a well-defined border.

### Comparative Analysis of Meniscus Injury Repair Experimental Groups

In this paper, a comparative analysis of the meniscus recovery state of the 6-weeks specimens of group A and group B is carried out, as shown in Tables 2, 3:

**TABLE 2:** Restoration state of meniscus in group A of specimens at 6 weeks.

	healing speed	Activity	metabolic rate
1	20%	30%	18%
2	21%	35%	21%
3	24%	39%	23%
4	27%	40%	24%
5	28%	42%	26%
6	29%	43%	29%

**TABLE 3:** Restoration state of meniscus in group B of specimens at 6 weeks.

	healing speed	Activity	metabolic rate
1	29%	30%	25%
2	36%	33%	29%
3	43%	40%	38%
4	58%	45%	47%
5	60%	56%	65%
6	66%	60%	70%

As shown in Tables 2, 3: Injury to the avascular area of the meniscus is incurable by itself. It can be repaired by transplanting fibrochondrocytes and fibrogel complexes, fibrogel can repair the meniscus tissue and promote the healing of meniscus damage. It not only provides a scaffold for cell movement and proliferation, but also provides a cytokine depot by maintaining cells and a nutrient solution, allowing fibrochondrocytes to survive and proliferate at the edge of the meniscus injury. Finally, the healing process of the meniscus is completed with the disappearance of protein breakdown.

## Conclusion

The meniscus of the knee is the most vulnerable to injury during sports and everyday life. In the past, due to the underdeveloped medical technology, after the meniscus of the knee was damaged, there was no suitable treatment method. However, with the development of society, the improvement of technology, and the development of today’s science and technology, the treatment of knee meniscus injury has also improved, and it can be judged and diagnosed from multiple angles. In daily life and sports activities, the meniscus in the knee joint functions as a load-bearing transmission, absorbs shock, maintains the overall stability of the knee joint of the foot, and helps lubricate the joint. Because the burden on the human body is relatively large, the damage will also be very large. Therefore, this paper conducts a specific analysis around tissue engineering nanomaterials and meniscus sports injury, and proposes a sports rehabilitation method based on the Kalman filter theory in the method part. This introduction is relatively clear. In the experimental part, the experiment was carried out on eight rabbits with meniscus injury, and then divided into two groups. The experimental group that added nanomaterials recovered significantly faster than the experimental group that simply allowed it to heal. It can be seen that tissue engineered nanomaterials can promote the repair of meniscus sports injuries. However, due to the limited ability of the author, the experiment in this paper is not very realistic. In order to further improve the validity of the experiment, more detailed studies on a large number of samples, biomaterials, biomechanics and biochemistry are required. Due to the limited professional ability of the author, there are many negligence in the experiment, especially the data may cause differences due to the experimental environment. The author will continue to study the role of nanomaterials in meniscal injury repair. Strive to do better.

## Data Availability

The original contributions presented in the study are included in the article/supplementary material, further inquiries can be directed to the corresponding author.
